# Multifunctional photodynamic/photothermal nano-agents for the treatment of oral leukoplakia

**DOI:** 10.1186/s12951-022-01310-2

**Published:** 2022-03-04

**Authors:** Lin Lin, Chuanhui Song, Zheng Wei, Huihui Zou, Shengwei Han, Zichen Cao, Xinyu Zhang, Guorong Zhang, Jianchuan Ran, Yu Cai, Wei Han

**Affiliations:** 1 Department of Oral Medicine, Nanjing Stomatological Hospital, Medical School of Nanjing University, 30 Zhongyang Road, Nanjing, 210008 China; 2 Department of Oral and Maxillofacial Surgery, Nanjing Stomatological Hospital, Medical School of Nanjing University, No 30 Zhongyang Road, Nanjing, 210008 China; 3 Center for Rehabilitation Medicine, Rehabilitation & Sports Medicine Research Institute of Zhejiang Province, Department of Rehabilitation Medicine, Zhejiang Provincial People’s Hospital (Affiliated People’s Hospital, Hangzhou Medical College), Hangzhou, 310014 Zhejiang China; 4 Central Laboratory of Stomatology, Nanjing Stomatological Hospital, Medical School of Nanjing University, No 30 Zhongyang Road, Nanjing, 210008 China; 5 Institute of Translational Medicine, The Affiliated Drum Tower Hospital of Nanjing University Medical School, Nanjing, 210008 China; 6 Pediatric Dentistry, Nanjing Stomatology Hospital, Medical School of Nanjing University, No 30 Zhongyang road, Nanjing, 210008 China

**Keywords:** Nanomedicine, Photodynamic therapy, Oral leukoplakia, Tumor

## Abstract

Oral leukoplakia (OLK) has gained extensive attention because of the potential risk for malignant transformation. Photosensitizers (PSs) played an indispensable role in the photodynamic therapy (PDT) of OLK, but the poor light sensitivity greatly hampered its clinical application. Herein, a novel organic photosensitive ITIC-Th nanoparticles (ITIC-Th NPs) were developed for OLK photodynamic/photothermal therapy (PTT). ITIC-Th NPs present both high photothermal conversion efficiency (~ 38%) and suitable reactive oxygen species (ROS) generation ability under 660 nm laser irradiation, making them possess excellent PDT and PTT capability. In 4-nitroquinoline 1-oxide (4NQO)-induced oral precancerous animal models, ITIC-Th NPs effectively suppress the OLK's cancerization without apparent topical or systemic toxicity in vivo. This study offers a promising therapeutic strategy for PDT and PTT in OLK treatment, and this study is the first interdisciplinary research in the field of multimodal therapy for OLK.

**Graphical Abstract:**

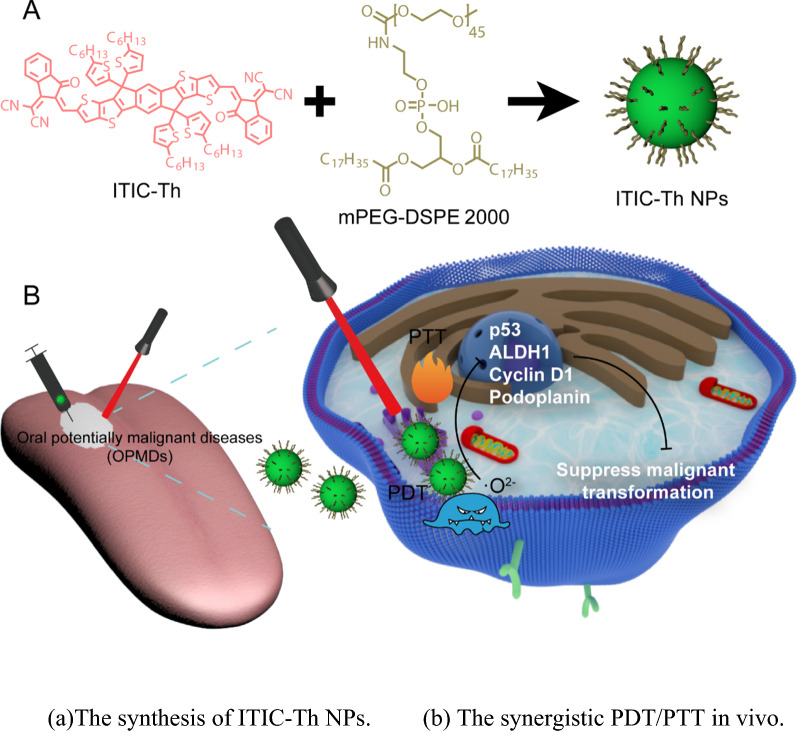

**Supplementary Information:**

The online version contains supplementary material available at 10.1186/s12951-022-01310-2.

## Introduction

Oral squamous cell carcinoma (OSCC) accounts for more than 90% of oral malignancies, and most OSCC develops from oral precancerous lesions, which are collectively referred to as potentially malignant disorders (OPMDs) [1, 2]. According to the definition of the World Health Organization (WHO), oral leukoplakia (OLK) is one of the most common OPMDs in oral mucosa, which is now defined as white irreversible and non-scratchable plaques that are at risk of turning into cancer [3, 4]. To date, the prevalence rate of OLK is 3.5% (range: 0.13–34.0%), among which 4–13% could be transformed into malignant disease [5, 6]. The presence and grading of epithelial dysplasia are the most commonly used prognostic factors to predict malignant transformation, which might be significantly reduced but not eliminated after the excision. Thus, OLK is an excellent clinical model for examining cancer prevention strategies [7–9].

Photodynamic therapy (PDT), as a minimally invasive approach, plays an indispensable role in the management of OLK. Unlike surgical resection, PDT is highly selective and repeatable with minimal scarring and is particularly valuable in regions with underlying functional structures [10, 11]. 5-aminolevulinic acid (5-ALA) is a water-soluble photosensitizer widely used to treat OPMDs, especially OLK. It can be administered intravenously, orally, or locally [12]. It is a precursor of porphyrins, which can't produce reactive oxygen species (ROS) by itself. After exogenous 5-ALA enters cells, it converts to porphyrin IX (PpIX) through the porphyrin-haem pathway, ROS generated once the PpIX is exposed to visible light (including 400–410 nm and 635 ± 5 nm) [13]. Moreover, 5-ALA-based PDT should be administered every 2–3 weeks, and repeated treatment is usually required to achieve desired results. Significantly, the treated areas should be strictly protected from light for 48 h after PDT, and this exposure prophylaxis would continue throughout the entire treatment. Once inadvertently exposed to sunlight or even indoor light, papules, macules, blisters, or erosion may occur in the PDT-treated areas [14]. In addition, after the treatment, the lesions need to be protected from saliva for more than 3 h with thick gauze, which would restrict the patients' movement to a great extent [15, 16]. According to the literature, the recurrence rate of 5-ALA-based PDT is as high as 60% [14, 17, 18]. Therefore, more efficient and fewer side effect photosensitizers should be developed to better study the PDT treated OLK.

The increasing cross-research between biomedicine and nanoscience provides more opportunities to develop new photosensitive materials for clinical treatment [19, 20]. Near-infrared (NIR) phototherapy has been widely applied for biomedicine due to its deep tissue penetration, robust anti-tumor efficacy, lower drug resistance, high specificity, and minimal invasiveness [21, 22]. In particular, photothermal therapy (PTT) is the other phototherapeutic method, which refers to light radiation and systemic local spot hyperthermia to heat the disaffected cells locally [23]. PTT can cause protein denaturation and cell membrane rupture, leading to irreversible damage to the target lesions [23–25]. To conquer the limitations of 5-ALA-based PDT, integrating PDT/PTT into OLK can block the cancerization process, promote the extinction of OLK lesions, and truly achieve precision medicine.

Herein, a novel acceptor–donor-acceptor (A-D-A) organic photosensitive nano-agents based on 3,9-bis(2-methylene-(3-(1,1-dicyanomethylene)-indanone))-5,5,11,11-tetrakis-(5-hexylthienyl) dithieno[2,3-d:2’,3’-d’]-s-indaceno[1,2-b:5,6-b’]dithiophene (ITIC-Th) is designed for PDT/PTT. ITIC-Th is an efficient fused-ring electron acceptor with an alkyl thiophene group chain, making excellent NIR absorption and emission [26, 27]_._ By nanoprecipitation, we successfully prepared ITIC-Th nanoparticles (NPs) with a J-aggregative state, which present the advantages of redshift absorption, water solution, nontoxicity, and biodegradation ability. ITIC-Th NPs possess high photothermal conversion efficiency (~ 38%) and high ROS generation ability, endowing them with phototherapy both in vitro and in vivo. After studying the optical properties in vitro, we proved the local and system biosafety and excellent phototherapeutic efficacy. Furthermore, we successfully established a dynamic precancerous animal model similar to humans in pathogenesis, pathological changes, host immune activity, and molecular level. The in vivo study demonstrated that the malignant transformation could be effectively blocked through phototherapy. In this study, the multifunctional organic nano-agents were applied in the PDT/PTT treatment of OLK for the first time, and the established treatment mode was much close to the real clinical practice, which has a broad application prospect in treating oral precancerous lesions.

## Methods

### Materials

ITIC-Th was purchased from Derthon (Shenzhen, China). Tetrahydrofuran (THF) and dichloromethane were purchased from Admas-Beta (Shanghai, China). DSPE-mPEG 2000 was purchased from ToYongBio Tech (Shanghai, China). Leuk-1 was kindly provided by Professor Li Mao of the University of Maryland Dental School (Baltimore, USA). CAL 27 and HaCaT cell lines were get from the American Type Culture Collection (ATCC; Manassas, VA, USA). Singlet Oxygen Sensor Green (SOSG) kit was from Invitrogen (Thermo Fisher Scientific, USA). 1, 3-diphenylisobenzofuran (DPBF) and 4-nitroquinoline 1-oxide (4NQO) were purchased from Sigma-Aldrich (Saint Louis, USA). Cell Counting Kit-8 (CCK-8) was purchased from Bimake (Shanghai, China). ROS assay kit and DAPI were obtained from Beyotime (Shanghai, China). Cyclin D1 Monoclonal Antibody, p53 Monoclonal Antibody, Aldehyde dehydrogenase isoform 1 (ALDH1) Polyclonal Antibody, Programmed cell death 5 (PDCD5) Polyclonal antibody, and β-Actin Monoclonal Antibody were purchased from Proteintech (Wuhan, China). The anti-podoplanin (PDPN) antibody was purchased from Abcam (Shanghai, China). 5-ALA was purchased from FUDAN-ZHANGJIANG (Shanghai, China). Paraformaldehyde was from BOSTER (Wuhan, China). The Super -maxvision mouse/rabbit universal HRP Kit was from Typing Biotech (Nanjing, China).

### Synthesis of ITIC-Th NPs

**Fig. 1:**
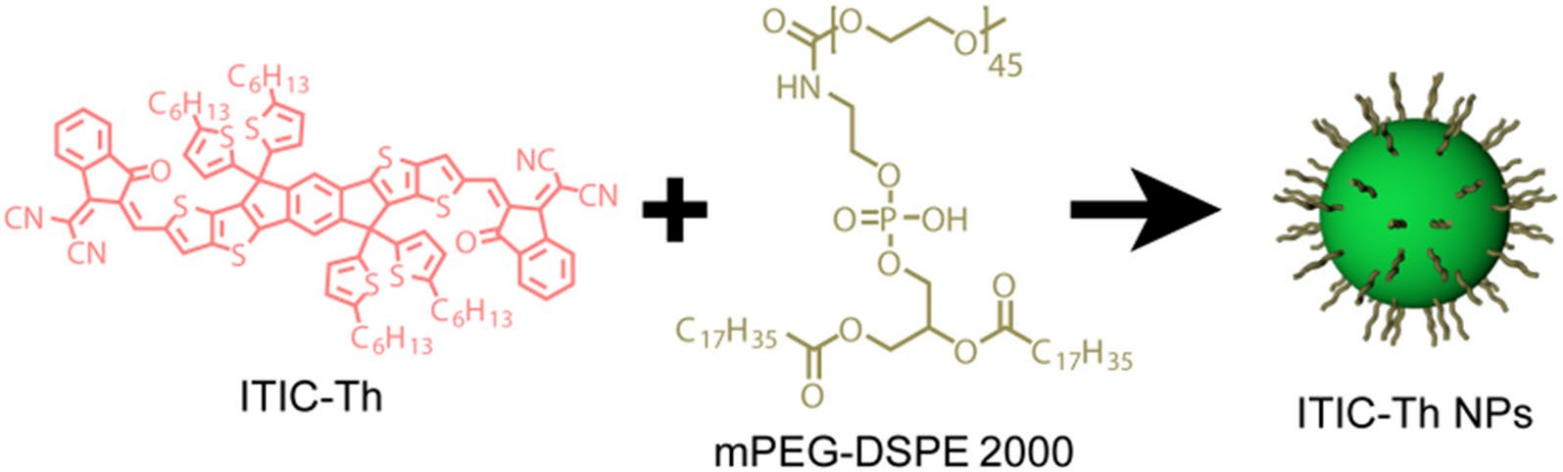
The illustration of the synthesis of ITIC-Th NPs

Referring to the previous literature, ITIC-Th NPs were synthesized by the reprecipitation method [28]. Briefly, under ultrasonic oscillation, 2 mL of ITIC-Th solution (250 μg/mL, THF) was dripped into the vigorously stirred DSPE-mPEG 2000 solution (1 mg/mL, ddH_2_O) slowly (Fig. 1). After the solution was clear and transparent, THF organic solvent was removed and purified by rotary evaporation to obtain water-soluble ITIC-Th NPs.

### Characterization of ITIC-Th NPs

The ITIC-Th NPs morphology was observed on a transmission electron microscope (TEM, Hitachi H-7600, Japan), and the hydrodynamic diameter was measured by a dynamic light scattering system (DLS, BT-90). The UV − vis spectrum was obtained by an UV − vis − NIR spectrophotometer (PerkinElmer LAMBDA 750), and the fluorescence spectrum was acquired by a photoluminescence spectrometer (FLS980, Edinburgh Instruments, UK). A NIR fluorescence camera detected the NIR fluorescence imaging. Different thickness beef covered NPs solutions were used to check the fluorescence penetration of NPs.

### Photothermal performance of ITIC-Th NPs

The photothermal performance of ITIC-Th NPs solution was measured through recording the solution (0, 6.5, 12.5, 25.0, and 50.0 μg/mL) temperature change with 660 nm laser irradiation (0.4, 0.6, 0.8, 1.0, and 1.2 W/cm^2^) by an IR-thermal camera (FLIR, Arlington, VA, E50), while PBS was used as control. The photothermal conversion efficiency (η) of ITIC-Th NPs was calculated by the Formula (1) [29]

1 $$\begin{array}{c}\eta =\frac{hA\left({T}_{M}-{T}_{S}\right)-{Q}_{Dis}}{I\left(1-{10}^{-A660}\right)}\end{array}$$

where *h* is the heat transfer coefficient, A represents the container surface area, T_M_ is the maximum temperature, T_S_ means the ambient temperature. Q_Dis_ is the heat dissipation, the I is the incident laser power, and A660 is the absorbance of the sample at 660 nm. The *h*A can be calculated from Formula (2)

2 $$\begin{array}{c}{\tau }_{s}=\frac{{m}_{D}{c}_{D}}{hA}\end{array}$$

where τ_s_ is the time constant for heat transfer of the system, calculated from Additional file 1: Fig S1; m_D_ and c_D_ are the mass and heat capacity, respectively, of the water. Q_Dis_ represents the heat dissipation from the light absorbed by the water and the quartz sample cell, so Q_Dis_ was calculated according to Formula (3)

3 $$\begin{array}{c}{Q}_{Dis}=\frac{{c}_{D}{c}_{D}\left({T}_{M\left(water\right)}-{T}_{S}\right)}{{\tau }_{s\left(water\right)}}\end{array}$$

where T_M (water)_ is the maximum water temperature under laser irradiation.

### Photodynamic performance of ITIC-Th NPs

The fluorescent probe SOSG and DPBF measured the generation of ^1^O_2_. ITIC-Th NPs (25 μg/mL, ddH_2_O) were mixed with SOSG solution and irradiated by a 660 nm laser (1.0 W/cm^2^) over 10 min. The fluorescence spectrum of oxidized SOSG (excitation/emission: 488/525 nm) was recorded by a UV vis NIR spectrophotometer every 10 s. ITIC-Th NPs (25 μg/mL, dichloromethane) and DPBF (2 μM) were dissolved into THF, then the solution was followed by irradiation (660 nm, 1.0 W/cm^2^), then immediately detected the UV–Vis absorption. The ^1^O_2_ quantum yield was evaluated by previous literature using Formula (2) [28].

$$\varphi (unkown) = \varphi \Delta \left( {ref} \right)\left[ {\frac{{k(unkown)}}{{k(ref)}}} \right]\left[ {\frac{{F(ref)}}{{F(unkown)}}} \right]\left[ {\frac{{PF(ref)}}{{PF(unkown)}}} \right]$$

where $unkown$ and ref are the 'ITIC-Th NPs' and 'MB', respectively. Quantum yields of singlet oxygen measured upon irradiation at MB as reference took into account as 0.52. k is the slope of the difference in change in absorbance of DPBF with the irradiation time. F is the absorption correction factor given by F = 1–10^−OD^ (OD at the irradiation wavelength), and PF is the laser power.

### Cell culture

Leuk-1, a premalignant leukoplakia cell line, was cultured in a defined keratinocyte serum-free medium (K-SFM; GIBCO, Invitrogen, Grand Island, USA). The OSCC cell line CAL 27 and human immortalized keratinocyte line (HaCaT) were cultured in a complete Dulbecco's modified Eagle's medium (DMEM, Key GEN Bio TECH, China) medium with 10% Fetal Bovine Serum (FBS, BI, Israel). These cells were cultured in an incubator at 37 °C with 5% CO_2_.

### Cell cytotoxicity assay

The CCK-8 assay detected the cytotoxicity of ITIC-Th NPs in Leuk-1 cells. Leuk-1 cells were seeded into a 96-well plate with different concentrations of ITIC-Th NPs and incubated for 24 h, then some of them were exposed to 660 nm laser (1.0 W/cm^2^) irradiation. Afterward, a CCK-8 assay was used to detect the cell viability of different groups. Another group of Leuk-1 cells was seeded in six-well plates and incubated overnight. The medium was replaced with fresh K-SFM containing ITIC-Th NPs or not. After another incubation for 12 h, cells were exposed to a 660 nm laser, and the cells were washed with PBS three times. Calcein-AM/PI Double Staining Kit labeled live cells in green and dead cells in red under the confocal microscope.

### Detection of intracellular ROS

Intracellular ROS level was detected using the ROS Assay Kit. 1 × 10^6^ Leuk-1 cells were planted into a 35 mm glass-bottomed dish overnight. The attachable cells were cocultured with ITIC-Th NPs for another 4 h, after washing with PBS, 2, 7-dichlorodi-hydrofluorescein diacetate (DCFH-DA) probe with the K-SFM medium at 1: 1000 was added to the dishes and incubated for 20 min, followed by 660 nm laser irradiation (1.0 W/cm^2^). Fluorescence was detected by the Nikon Digital Eclipse A1 Plus microscope.

### Western blot analysis

Proteins were extracted from the HaCaT /CAL 27/Leuk-1 cell. The primary antibodies used were p53, PDPN, ALDH1, Cyclin D1, and PDCD5. β-actin was used as the loading control. Protein levels were quantified using Image-Pro Plus and normalized to the β-actin level by densitometry.

### Patients

Ten patients undergoing surgical treatment for oral leukoplakia in the Department of Oral and Maxillofacial Surgery, Nanjing Stomatological Hospital (Jiangsu, China), from January 2018 to December 2019 were recruited to our study, half of them had epithelial dysplasia and the other half did not. Oral leukoplakia was clinically diagnosed and confirmed using histology. The specific clinical features are shown in Table S1. Informed consent was obtained from each patient. This study was approved by the Nanjing Stomatological Hospital Ethics Committee (Application Permit Number: 2019NL-029(KS)).

### Immunohistochemical staining and score

Immunohistochemistry was carried out according to the manufacturer's protocols. All immunostained sections were independently assessed and scored by two experienced pathologists using Imagescope software (Scanscope, USA) for quantitative analysis of images based on the visual evaluation. The related protein was quantified by visual grading according to the degree of staining: 0 (< 5%), 1 (6–25%), 2 (26–50%), 3 (51%-75%), 4 (> 75%), staining strength: 0 (no staining), 1 (weak staining), 2 (medium staining), 3 (strong staining). The scores were multiplied according to the intensity and degree of staining to obtain the final immune response score.

### Establishment of oral leukoplakia model in rats

A total of 28 female-specific pathogen-free Sprague–Dawley (SD) rats, six to eight weeks of age, were purchased from Beijing Vitalstar Biotechnology Co.,Ltd. All animal experiments were followed by the guidelines international and national approved by the Nanjing Stomatological Hospital Ethics Committee (Application Permit Number: 2019NL-029(KS)). After a week of acclimation, 26 rats were treated with 75 µg/ml 4NQO dissolved in propylene glycol in the drinking water, and the remaining two rats were treated with normal drinking water. One rat was sacrificed in the 8th and 12th weeks, respectively. The tongue was excised, cut longitudinally, and fixed with 10% buffer formaldehyde for 48 h. According to the manufacturer's protocols, the tissues were then paraffin-embedded, sectioned at 4 μm, and stained with hematoxylin and eosin (H&E). In the 16^th^ week, two 4NQO rats and two normal drinking rats were sacrificed to evaluate the progress of oral leukoplakia and the biosafety of 4NQO. The tongue, heart, liver, spleen, lung, and kidney were resected to perform histopathological analysis. The induction lasted for 16 weeks, with all rats drunk as needed and switched to purified water at week 17.

### Thermal imaging

Finally, twenty rats with typical white patches on the back of the tongue were selected and randomly separated into five groups (n = 4 per group, blank control, PBS/ALA with laser irradiation, ITIC-Th NPs with or without laser irradiation). The remaining two rats were excluded because of atypical leukoplakia or inappropriate location. The rats were anesthetized by 2% isoflurane inhalation, after complete sedation, a mouth gag was applied to open their mouth and take out their tongues. The first group received no injections, while Group 2 was intralesional injection with 100 μl PBS, Group 3 with 100 μl freshly prepared 20% 5-ALA, and Group 4/5 with the same dose of 100 μg/ml ITIC-Th NPs. Injections made from the marginal base of the target area and the scope should exceed 3–5 mm beyond the edge of the intended treatment site. In Group 1/2/5, the thermal imaging was measured by an infrared camera (FLIR, C3) at different time points following a 660 nm laser (1 W/cm^2^) irradiation for 3 min. While in the third group, a 365 nm UV light was performed 3 h post-injection to test the illumination reaction. Afterward, a laser at the wavelength of 632 nm (0.5 W/cm^2^) was also irradiated for 3 min. Therapies were conducted every five days and each rat received three treatments.

### Efficacy evaluation in vivo

The therapeutic effect was evaluated and independently scored by two experienced oral mucosal clinicians. Four parameters were assessed for each subject, including size, type, color, and texture [30, 31]. If the lesions extended to two or more sites, the site with more severe lesions was selected as the target area for evaluation. The size of the oral leukoplakia was determined by measuring any clinically visible area of morphological change and calculated as 'length × width'. The same investigator made measurements before the treatment (Day 0) and after complete treatment (Day 14), respectively. Before treatment, all initial areas were recorded as 0 points. On day 14, compared with the previous period, the cross-sectional area of measurable leukoplakia decreased by ≥ 50% was recorded as negative 2 points, increased by ≥ 50% was recorded as 2 points, and the rest of them were labeled as 0 points. The types of lesions are mainly divided into three grades according to surface roughness, protuberance degree, ulcer or erosion: granular protuberance, verrucous protuberance, villous protuberance or prickly protuberance, which are significantly higher than the mucosal surface, or those with ulcers are marked as 3 points; Uneven and higher than the mucosal surface, the local ulcer was marked as 2 points; Homogeneous plaques slightly above the mucosal surface without ulceration were scored as 1 point. Color is divided into two grades, pure white is marked as 2 points, gray or light white is marked as 1 point. The texture is graded on three scales, with 3 for hard, 2 for medium, and 1 for soft. The total score was calculated by adding up all the scores. Cases that are clinically evaluated as cancerous are scored directly as 10 points. All operations were performed under anesthesia. Finally, all of the rats were euthanized on the 14th day, tongues were collected, and stained by H&E. Immunohistochemistry (ALDH1 and p53) was carried out according to the manufacturer's protocols.

### Chronic toxicity experiment

The body weight was measured and recorded every 2 days. After rats were sacrificed, the tongues and their adjacent tissues, the major organs were gathered for histological analysis, and blood samples were collected for routine and biochemical examinations.

### Statistical analysis

All analyses were carried out as indicated by GraphPad Prism 8.0. Mean and standard deviation was used as descriptive statistics. The student's *t*-test was used to calculate statistical differences between two groups, and one-way analysis of variance (ANOVA) was used to compare differences between more than two groups. The value of p < 0.05 was considered statistically significant.

## Results and discussion

### Preparation and characterization

**Fig. 2:**
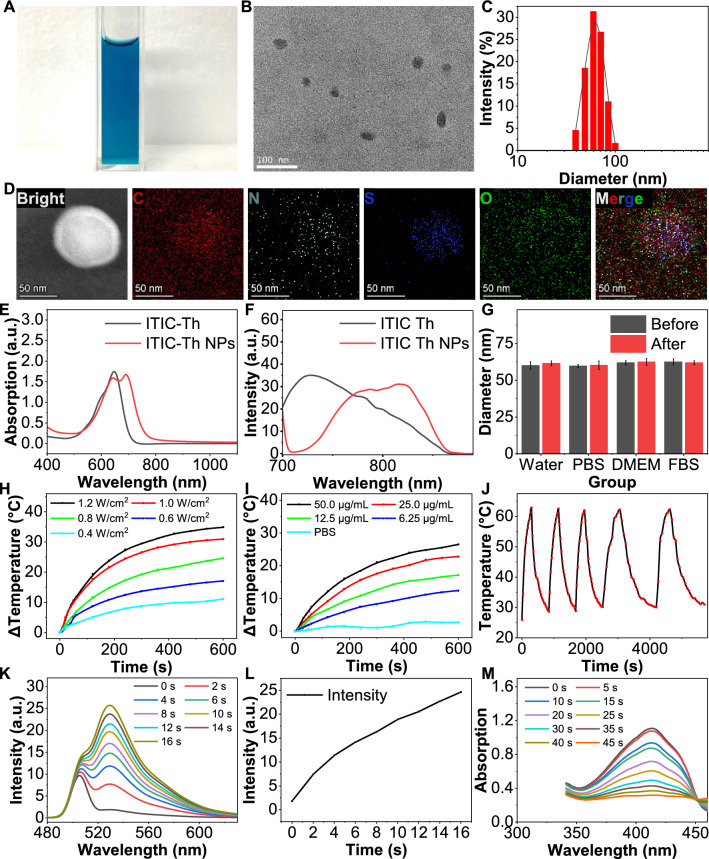
The characterization of ITIC-Th NPs. **A** Optical image of the ITIC-Th NPs water solution in a cuvette. **B** The TEM image of ITIC-Th NPs in water solution. Scale bar = 100 nm. **C** The DLS profiles of ITIC-Th NPs in water (10 μg/mL). The average diameters of ITIC-Th NPs were approximately 60 nm, consistent with the TEM result. **D** The corresponding energy-dispersive spectroscopy (EDS) results of ITIC-Th NPs (The corresponding elements were marked in different colors in the image). **E** UV absorbance spectrum of ITIC-Th and ITIC-Th NPs (20 μg/mL) solution. **F** The fluorescence emission spectrum of ITIC-Th and ITIC-Th NPs under emission of 660 nm. **G** The average diameter of ITIC-Th NPs in corresponding solutions (water, PBS, DMEM, and FBS) after 2 weeks. **H** Photothermal profiles of ITIC-Th NPs in PBS under different power density irradiation (660 nm, 0.4, 0.6,0.8,1.0, and 1.2 W/cm^2^). **I** Photothermal profiles of ITIC-Th NPs in PBS with different concentrations (6.25, 12.5, 25.0, and 50.0 μg/mL) under laser irradiation (660 nm, 1 W/cm^2^). **J** The heating curve of the ITIC-Th NPs in PBS during 5 cycles of on-and-off laser irradiation (660 nm, 1 W/cm^2^). **K** Fluorescence spectra of ITIC-Th (20 μg/mL) mixed with SOSG (10^–5^ mol/L) upon laser irradiation (660 nm, 1 W/cm^2^) with various duration. **L** Fluorescence intensity was measured at different time intervals. **M** Absorption spectra of ITIC-Th (20 μg/mL) mixed with DPBF (6 × 10^−5^ mol/L) under laser irradiation (660 nm, 1 W/cm^2^) with various durations

ITIC-Th NPs were synthesized by the nanoprecipitation method reported in our previous literature [28, 32] and had a uniform and transparent appearance in an aqueous solution (Fig. 2A). TEM images of ITIC-Th NPs presented a diameter of approximately 50 nm with spherical-like morphology (Fig. 2B), in diameter in favor of cellular uptake [33]. DLS results indicated that the average size of ITIC-Th NPs was nearly 60 nm, which was consistent with the TEM result (Fig. 2C). The element mapping analysis also showed the corresponding element distribution in the NPs (Fig. 2D), where the carbon (C), nitrogen (N), sulfur (S), and oxygen (O) were marked as red, cyan, blue, and green, respectively, indicating the success of synthesizing. The UV–Vis spectrum of free ITIC-Th exhibited peaks at 646 nm. When ITIC-Th NPs were formed, the peak showed a redshift to 692 nm, which confirmed the presence of NPs (Fig. 2E). The fluorescence spectra of ITIC-Th NPs showed that the emission peak was 820 nm (Fig. 2F), indicating the fluorescence imaging ability in the near-infrared [34]. To test the stability of NPs, we checked the particles' size and appearance in different solutions for two weeks [35]. The results showed that the as-prepared NPs had good stability (Fig. 2G and Fig S1A). Considering the microenvironment in OLK, we further checked the stability of NPs under different pH circumstances. The UV–VIS spectrum and DLS results showed a minimal difference between different pH, indicating that the property of NPs can stay stable regardless of the environment (Additional file 1: Fig S1B, C). All those data suggested that the NPs were stable enough for therapy in vitro and in vivo.

### Photothermal and photodynamic properties

The photothermal properties of the ITIC-Th NPs were evaluated by using a 660 nm laser as the excitation light. The photothermal effect of ITIC-Th NPs was positively correlated with the power density. The elevated temperature of ITIC-Th NPs (100 μg/mL) gradually increased from 11.1 °C to 34.9 °C at 10 min' irradiation (the power density from 0.4 to 1.2 W/cm^2^). Even at a low intensity of 0.8 W/cm^2^, the solution temperature was still lifted by 24.7 °C (Fig. 2H). The temperature variation curves of ITIC-Th NPs solutions were also positively correlated with concentration. Under continuous laser irradiation (1.0 W/cm^2^), the temperature of ITIC-Th NPs (50 μg/mL) could increase by 26.5 °C at 10 min, which demonstrated the excellent photothermal performance (Fig. 2I). The photothermal conversion efficiency (η) [29] of ITIC-Th NPs was calculated to be nearly 38.6% (Additional file 1: Fig S2A, B). Subsequently, we further examined the photothermal stability of ITIC-Th NPs, which is vital towards a photosensor [36]. We found that the maximum photothermal temperature of ITIC-Th NPs barely changed after five reversible heating and cooling cycles (Fig. 2J). This result highlighted the excellent photothermal stability of ITIC-Th NPs, which was beneficial for PTT application in vivo*.* To determine the singlet oxygen (^1^O_2_) production of ITIC-Th NPs, the fluorescent probe SOSG and DPBF were utilized, respectively. The intensity of ITIC-Th NPs and SOSG solution drastically increased under laser irradiation at 525 nm (660 nm, 1.0 W/cm^2^), indicating the amount of singlet oxygen generation. The singlet oxygen production of ITIC-Th NPs was 6.82% (Fig. 2K and L), which is close to that of free ICG (7.7%) used in the clinic [37]. The ROS generation ability of ITIC-Th NPs was further measured by using DPBF as a probe [38]. Figure 2M indicated the excellent ROS generation ability of ITIC-Th NPs. An electron spin resonance (ESR) test was conducted to analyze ROS species, which showed that the superoxide radical is the major radical oxygen species (Additional file 1: Fig S2C). The research results mentioned above demonstrated superior photothermal and photodynamic properties. During the treatment, the PDT-inducing cell dying can lead to the related antigen releasing. The PTT can increase local blood flow, promoting tissue healing and immune-related cells in vivo. Also, the cellular excessive ROS level could induce oxidative modification in other oxygen species, proteins, or lipids, even resulting in cellular damage and cell death [39], resulting in the relative cell markers alter.

### In vitro cytotoxicity

**Fig. 3:**
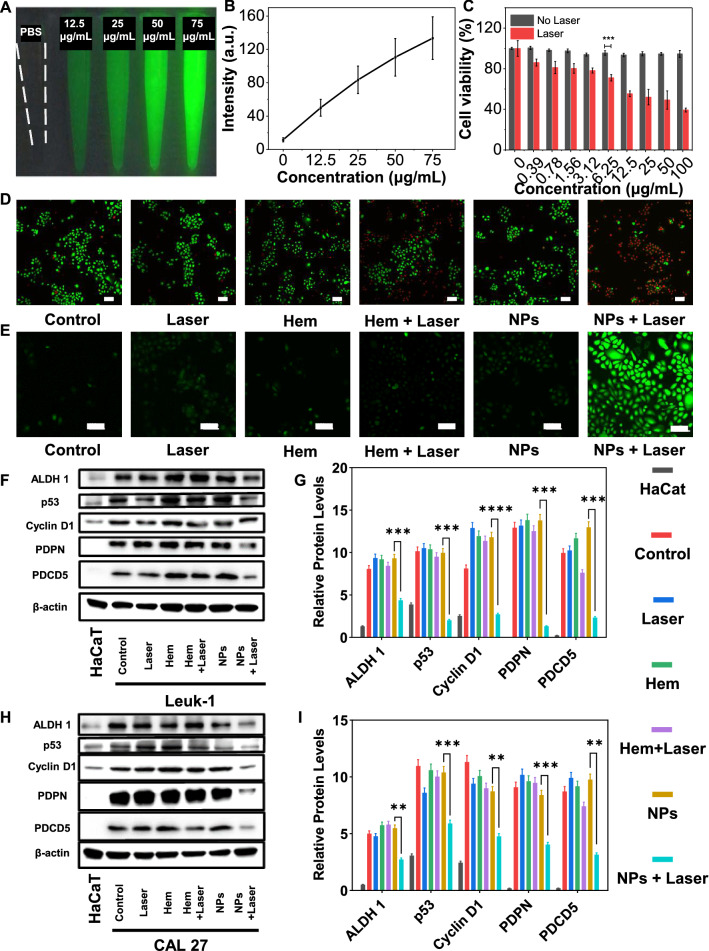
In vitro therapeutic evaluation of ITIC-Th NPs. **A** Fluorescence images of ITIC-Th NPs with a different concentration in PBS (Excitation wavelength: 660 nm). **B** Relationship between concentration of ITIC-Th and NIR fluorescence intensity. **C** CCK-8 evaluated the cell cytotoxicity assay. Leuk-1 cells were treated with various formulations for 24 h. **D** The fluorescence of Live/Dead cells. Live and dead cells were signaled in green and red fluorescence, respectively. Scale bar = 100 μm. **E** Fluorescence photographs of cellular ROS generation treated with ITIC-Th NPs (10 μg/mL). Scale bar = 100 μm. **F** Representative immunoblots indicated different protein levels in CAL 27 from each treatment group, and the quantitative analysis was shown in G. **H** Representative immunoblots indicated different protein levels in Leuk-1 from each treatment group, and the quantitative analysis was shown in I (**p < 0.01; ***p < 0.001)

The NIR imaging was used to determine the fluorescence imaging ability of ITIC-Th NPs. In vitro fluorescence imaging showed that pure PBS had no imaging capability, and the signal was generated with the addition of ITIC-Th NPs (Fig. 3A). Moreover, the fluorescence intensity increased with the increasing concentration and presented the brightest signal when the concentration reached 75 μg/mL compared to the background, proving that the ITIC-Th NPs had good imaging capability (Fig. 3B). Encouraged by the excellent fluorescence imaging property, we further tested the penetration ability of ITIC-Th NPs solution in NIR. Fluorescence imaging was used to detect penetration by covering different beef over the NPs solution. Even when the tissue thickness was increased to 4 mm, the NIR imaging can clearly show the contour of the capillary, suggesting the excellent tissue penetration of NPs (Additional file 1: Fig S3). The cytotoxicity of ITIC-Th NPs towards Leuk-1 cells in vitro was confirmed by CCK-8 assays in the presence or absence of 660 nm laser irradiation. Interestingly, ITIC-Th NPs were non-toxic to human keratinocytes HaCat and other normal cell lines (RAW 264.7 and THP-1) (Additional file 1: Fig S4A–C). Still, in premalignant leukoplakia cells, cell viability was suppressed dramatically under laser contrast to the no laser illumination group (Fig. 3C). IC_50_ values were calculated using GraphPad Prism 8.0, compared with hematoporphyrin (Additional file 1: Fig S4D), ITIC-Th NPs (IC_50_ = 10.36 μg/mL) showing a more apparent inhibitory effect on cell activity, implying the highly desirable PDT/PTT effect of ITIC-Th NPs, which was an effective method to avoid the potential drug side effects. Accordingly, 10 μg/mL was selected as the working concentration for subsequent experiments. To better analyze the cellular uptake of NPs, we used fluorescent PEG to visualize the particles in cells. The fluorescence showed the particles could distribute in the cells along the time (Additional file 1: Fig S5). The mechanism of cell death was further analyzed by FITC-Annexin V/ propidium iodide staining. ITIC-Th NPs were cultured in Leuk-1 cells at the concentration of 10 μg/mL with or without laser illumination (660 nm, 1 W/cm^2^), and non-treated cells were taken as control. The results observed by confocal laser microscopy were shown in Fig. 3D. Live cells were stained with Calcein-AM to show a green fluorescence signal, while dead cells were stained with PI to show red fluorescence signal. The cells were green in control, NPs, hematoporphyrin (Hem), laser group, meaning that the cells were sound and the NPs were safe without laser irradiation. After laser illumination, the ratio of cells with red signal was increased dramatically in the NPs group, suggesting the effective killing rate compared with the Hem group. The quantitative analysis was demonstrated in Additional file 1: Fig S6A, which consists of fluorescent images. All these data showed that the NPs possessed excellent biocompatibility without laser application but had a better therapeutic effect under laser irradiation than hematoporphyrin.

### ITIC-Th NPs inhibited OLK carcinogenesis in vitro

The generation of ROS plays a crucial role in treating OLK with PDT. Based on the efficient ROS generation ability in solution, we used the 2ʹ,7ʹ-dichlorofuorescin diacetate (DCFH-DA) probe to test the intracellular ROS generation in different groups and analyzed by CLSM and Image J [40]. DCFH-DA has no fluorescence, but it can be rapidly converted into fluorescent 20, 70-dichlorofluorescein by ROS [41]. As shown in Fig. 3E, a strong green fluorescence could be observed in Leuk-1 cells incubated with ITIC-Th NPs after laser irradiation (660 nm, 1 W/cm^2^). In contrast, negligible hematoporphyrin was emitted without ITIC-Th NPs or laser, suggesting the NPs produced much ROS after laser irradiation [42]. Furthermore, taking the hematoporphyrin with laser as a compared group, the green fluorescence is much more than that, indicating the effective ROS generation. The quantitative result in Additional file 1: Fig S6B further supported this conclusion (P < 0.01). These results proved that ITIC-Th NPs promoted a remarkable PDT therapeutic effect.

As high intracellular ROS levels can induce cell damage and even death, we speculate this may change the relative markers. To date, some biological biomarkers have been reported of potential value in predicting malignant transformation of oral leukoplakia, such as ALDH1, p53, Cyclin D1, PDCD5, and PDPN [43–45]. ALDH1, an isomer of aldehyde dehydrogenase, has been demonstrated to be a tumor stem cell marker for various solid tumors, including OSCC, and can be used as a prognostic marker for head and neck squamous cell carcinoma (HNSCC) survival [46]. p53 was known as a tumor suppressor gene that induced cell apoptosis, necrosis, and autophagy under cell stress. The mutation and inactivation of p53 were crucial in developing OLK [47]. Cyclin D1, a regulator of Cyclin-dependent kinase (CDK), promotes cell cycle progression during the G1 phase, which played a key role in cell proliferation and growth regulation, mitochondrial activity modulation, DNA repair, and cell proliferation migration control [48]. PDCD5 can be involved in programmed cell death, cell cycle, embryonic development, and immune regulation. The expression rate of PDCD5 deletion was consistent with the positive expression rate of mutant p53 and was decreased in laryngeal squamous cell carcinoma but still unclear in OSCC [49, 50]. PDPN was a mucin-type transmembrane glycoprotein, and positive expression of ALDH1/p53/Cyclin D1/PDPN was significantly associated with malignant transformation of OLK [51, 52]. As shown in Fig. 3F–I, the ALDH1/p53/ Cyclin D1/PDPN/PDCD5 protein expression levels significantly decreased after co-cultured with ITIC-Th NPs and laser irradiation (660 nm, 1 W/cm^2^). In particular, this phenomenon was more outstanding in the human tongue cancer cells than in the leukoplakia cells. These results indicated that ITIC-Th NPs could significantly down-regulate the expression of tumor-related genes in vitro. Since these biomarkers were associated with the risk of OLK's malignant transformation, we speculated that with photothermal and photodynamic effects, ITIC-Th NPs may prevent oral precancerous lesions from transforming into OSCC, although the specific mechanism still needs further studies.

To further determine the meaning of relative protein in oral leukoplakia, we detected the expression levels of these biomarkers in oral leukoplakia tissues with different levels of epithelial dysplasia. A total of 10 OLK patients (3 males and 7 females, mean age 59.6 ± 14.1 years) were enrolled in the study. Five of them had epithelial dysplasia, and the remaining did not. Compared with simple hyperplastic tissues, the ALDH1/p53/Cyclin D1/PDPN protein expression was more obvious in the tissues with epithelial dysplasia, which was significantly associated with malignant transformation of OLK. The role of PDCD5 in the development of OLK remains to be elucidated. In our research, PDCD5 expression was decreased with increasing histological grade, which was consistent with ovarian epithelial cancer findings, suggesting that it may also play an important role in OLK carcinogenesis (Additional file 1: Fig S7).

### Successful establishment of the oral leukoplakia rat model

**Fig. 4:**
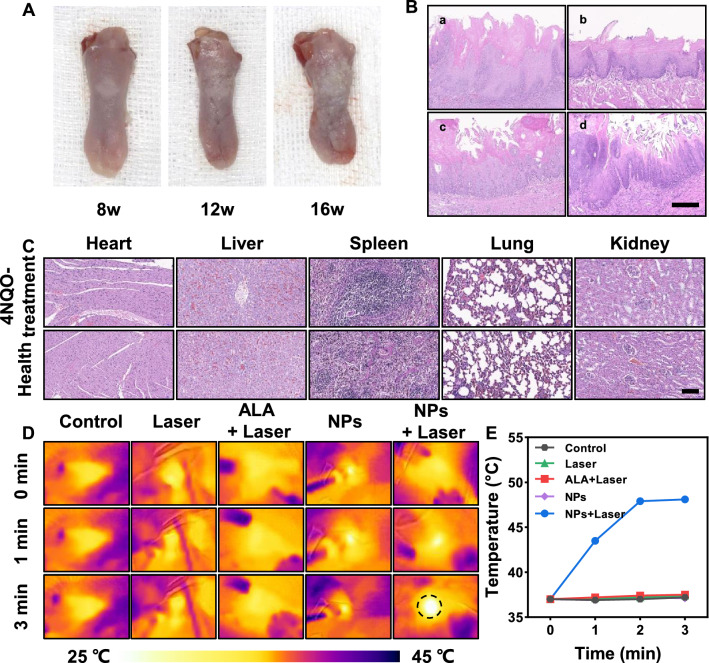
Induction of oral dysplastic lesions. **A** Representative images of isolated tongues at different administration time points. **B** Representative images of H&E staining of tongue tissue specimens of SD rats at week 16, including **a** Simple oral leukoplakia, **b** Oral leukoplakia with mild dysplasia, **c** Oral leukoplakia with mild-to-moderate dysplasia, **d** Oral leukoplakia with severe dysplasia (Scale bar = 200 μm). **C** H&E-stained histological images of major organs were collected after 16 weeks with/without 4NQO treatment (Scale bar = 200 μm). **D** In vivo infrared thermal images of rats under laser irradiation at different time intervals after intralesional injection with PBS/ALA/ITIC-Th NPs. Circles indicate the oral leukoplakia area at the base of the tongue. **E** The tongue's temperature evolution was measured in (**D**). (n = 4, mean ± SD)

4NQO is an aromatic amine heterocyclic compound widely used as the most recognized chemical precursor carcinogen [14]. Research has certified that 4NQO establishes precancerous or cancerous models by causing intracellular oxidative stress, DNA adduction, mutagenesis, and tumor induction [53, 54]. 4NQO-induced precancerous model is a multi-stage dynamic and continuous process from normal oral mucosa to OLK until OSCC, and pathological stages underwent hyperplasia, dysplasia (mild, moderate, and severe), carcinoma in situ, and invasive SCC [55, 56]. This model closely mimics the carcinogenesis of human oral dysplasia that is suitable for studying the specific cancerous process of OLK, especially in the development of biomarkers for early diagnosis and epithelial cell transformation [55, 57]. After feeding at 75 µg/ml 4NQO drinking water, all rats were anesthetized by isoflurane inhalation at 8, 12, and 16 weeks [58]. Researchers pulled outward the tongues to observe the changes, the typical tissue was chosen for histopathological examination. In the 8th week, the tongues remained normal, while in the 12th week they started to turn white. From this point on, all of the rats gradually exhibited non-detachable white plaque, and typical lesions appeared at week 16 similar to human oral leukoplakia (Fig. 4A). 4NQO has been proved to induce cancer in many parts of the oral cavity, such as the dorsal tongue, ventral tongue, palate, and even esophagus [53]. Many studies have confirmed that tongue lesions occurred almost 100%, especially on the dorsal mucosa. In our study, lesions localized mainly on the tongue dorsum with a few lesions on the palate or along the lateral borders of the tongue, which was consistent with the previous reports [53]. In the 16th week, we picked two 4NQO-treated rats at random and found varying degrees of pathological changes in tongue lesions, including simple oral leukoplakia and oral leukoplakia with mild, mild-to-moderate, or severe epithelial dysplasia (Fig. 4B). Also, the two normal drinking rats were sacrificed. Figure 4C showed no significant difference in histopathological features of major organs between 4NQO-treated and healthy rats. All the results above indicated that we had successfully established the rat model of oral leukoplakia, and 4NQO did not cause significant damage to the main organs in rats, and the rats can be used for further in vivo experiments.

### In vivo PTT/PDT of ITIC-Th NPs against oral leukoplakia

To explore the therapeutic effect of ITIC-Th NPs on oral leukoplakia in vivo and compared with the previous photosensitizer, we selected twenty rats with typical white plaque lesions on the dorsal surface of the tongue and randomly divided them into five groups (n = 4) for PTT/PDT. The rats received intralesional injections of 100 μg/ml ITIC-Th NPs or 20% 5-ALA and were irradiated with/without 660/632 nm laser for 3 min [14]. In clinical practice, doctors often choose gauzes soaked with the photosensitizer solution to reduce needle insertion pain to place over the lesion, and patients should minimize oral activity. Since we could not keep the rats' tongues inactive for three hours, the intralesional injection was applied to reduce the influence of salivary secretion or tongue movement on ALA absorption. The schedule was shown in Additional file 1: Fig S8. Rats without drug injections were used as blank controls, and rats injected with PBS were used as negative controls. The efficient accumulation of particles in pathological tissues is the key to PDT/PTT therapy. As the treatment schematic illustration is shown in Additional file 1: Fig. S8, local injection ensures direct access of NPs to the diseased tissue and local accumulation of solution after injection. A thermal monitor was used to observe the photothermal capacity of ITIC-Th NPs in vivo at different time points. The lesions' temperature of ITIC-Th NPs + laser group rapidly increased and reached about 48.1 °C, suggesting the superior photothermal conversion of such ITIC-Th NPs. Pure ITIC-Th NPs did not heat up in the absence of light, and ALA had a photodynamic effect but no photothermal effect, so the temperature of the other four groups had hardly changed (Fig. 4D, E). The NPs can work with PDT and PTT, which owe better therapeutic effects than ALA.

**Fig. 5:**
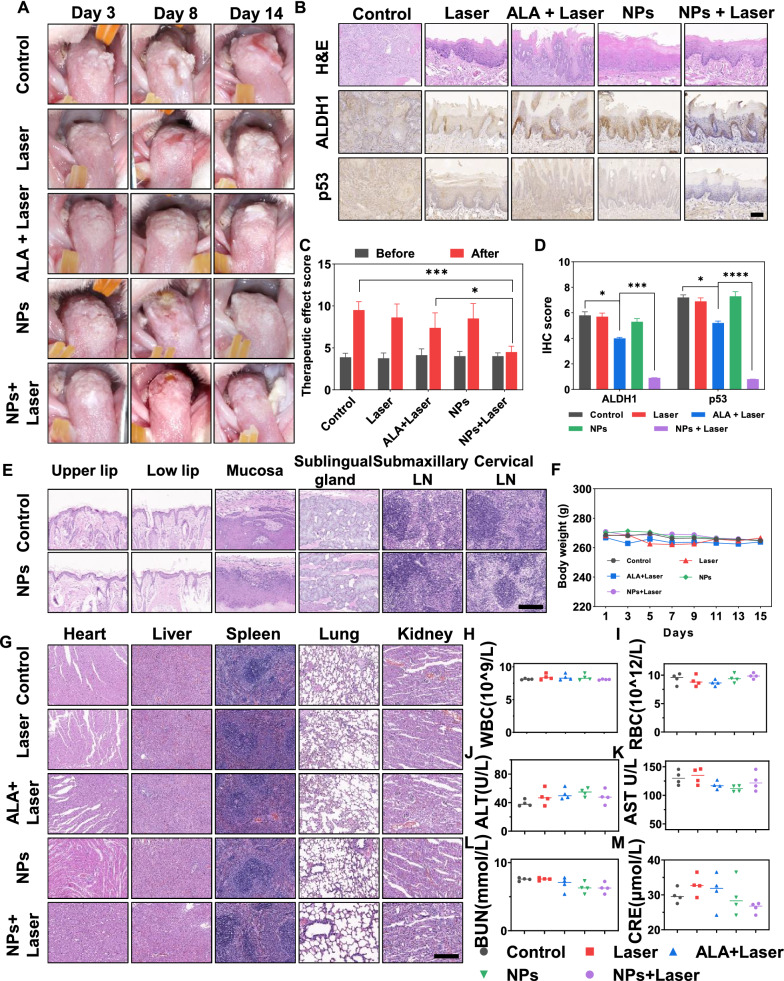
ITIC-Th NPs therapy intercepts the cancerous progression of oral leukoplakia in 4NQO rats. **A** Manifestations of tongue lesions in rats on days 3, 8, and 14 after different treatments. **B** Representative histological images of H&E, ALDH1, and p53 staining of collected tongue tissues. Scale bar = 200 μm. **C** Therapeutic effect scores of each group. According to lesion area, type, color, and texture, comprehensive judgment was made by two experienced oral mucosal clinicians. (n = 4, mean ± SD). **D** The score of ALDH1 and p53 IHC stain in B. (n = 4, mean ± SD, *p < 0.05, ***p < 0.001). **E** H&E-stained histological images of major tissues near the site of treatments. Scale bar = 200 μm. **F** Average body weight of different groups of rats. (n = 4, mean ± SD). **G** H&E-stained histological images of major organs after the different treatments. Scale bar = 200 μm. **H**–**M** Serum biochemical study and hematology assay after the different treatments. (n = 4, mean ± SD)

All operations above were performed by the same operator, conducted every five days and each rat received three treatments. After treatment, clinical, histological, and biological indicators were assessed. During the next 14 days, changes in tongue lesions were monitored and recorded. Two experienced oral mucosal clinicians evaluated the therapeutic effect independently, and the particular method was described above. None of our cases had achieved complete clinical response (CR, mean disappearance of all measurable disease). We speculated this was due to the continuous accumulation of 4NQO in the body [59, 60]. The dysplasia continued to worsen even though the 4NQO drinking water was switched back to purified water. This phenomenon could be verified in the blank control group (Table S2). The negative controls showed a faster oral leukoplakia cancerization rate, proving that only laser irradiation had no therapeutic effect. Otherwise, pure injection of ITIC-Th NPs showed limited cancerization inhibition, indicating a poor therapeutic effect of the NPs alone. According to meta-analysis, ALA-PDT's overall response rate (including complete and partial response) to OLK was estimated at 76.1% [10]. In our study, the therapeutic effect of ALA-PDT was slightly better than that of the control group. In contrast, for the ITIC-Th NPs group with 660 nm laser irradiation, the progression of carcinogenesis was almost controlled, and in several cases, the degree of keratosis was reversed due to the combined PTT and PDT effects, which showed high inhibition efficiency compared to ALA-PDT only (Fig. 5A and C). Furthermore, the immunohistochemical studies of tongue oral leukoplakia sections also highly supported the anti-carcinogenesis effects of various treatments, consistent with in vitro studies (Fig. 5B and D). The highest expression of ALDH1 and p53 was observed in the control group. In contrast, after being treated with ITIC-Th NPs + 660 nm laser, ALDH1 and p53 expression were significantly down-regulated. Therefore, the ITIC-Th NPs held superior anti-carcinogenesis efficacy.

In addition to the medical effectiveness of phototherapy, the long-term safety of photosensitizer must be considered. The biotoxicity of drugs was crucial for future biomedical application, so we conducted the biocompatibility of ITIC-Th NPs with SD rats. There was no apparent damage to the nearby vital tissues (upper lip, low lip, mucosa, sublingual gland, submaxillary lymph node (LN), and cervical LN, Fig. 5E), indicating the excellent biosafety of NPs. After intralesional injection of ITIC-Th NPs and laser irradiation, the body weight was recorded every other day. Each group did not have meaningful weight changes during the treatment (Fig. 5F). Major organs (heart, liver, spleen, lung, and kidney) were collected for H&E staining, and no noticeable pathological changes were observed (Fig. 5G). Blood samples were obtained from the eyes of SD rats. The results of blood routine, liver function, and kidney function indexes showed no differences among the groups, suggesting the safety of this therapy (Fig. 5H–M). Given all the evidence above, the ITIC-Th NPs had superior biosafety and displayed an extraordinary capability against oral leukoplakia carcinogenesis.

**Fig. 6:**
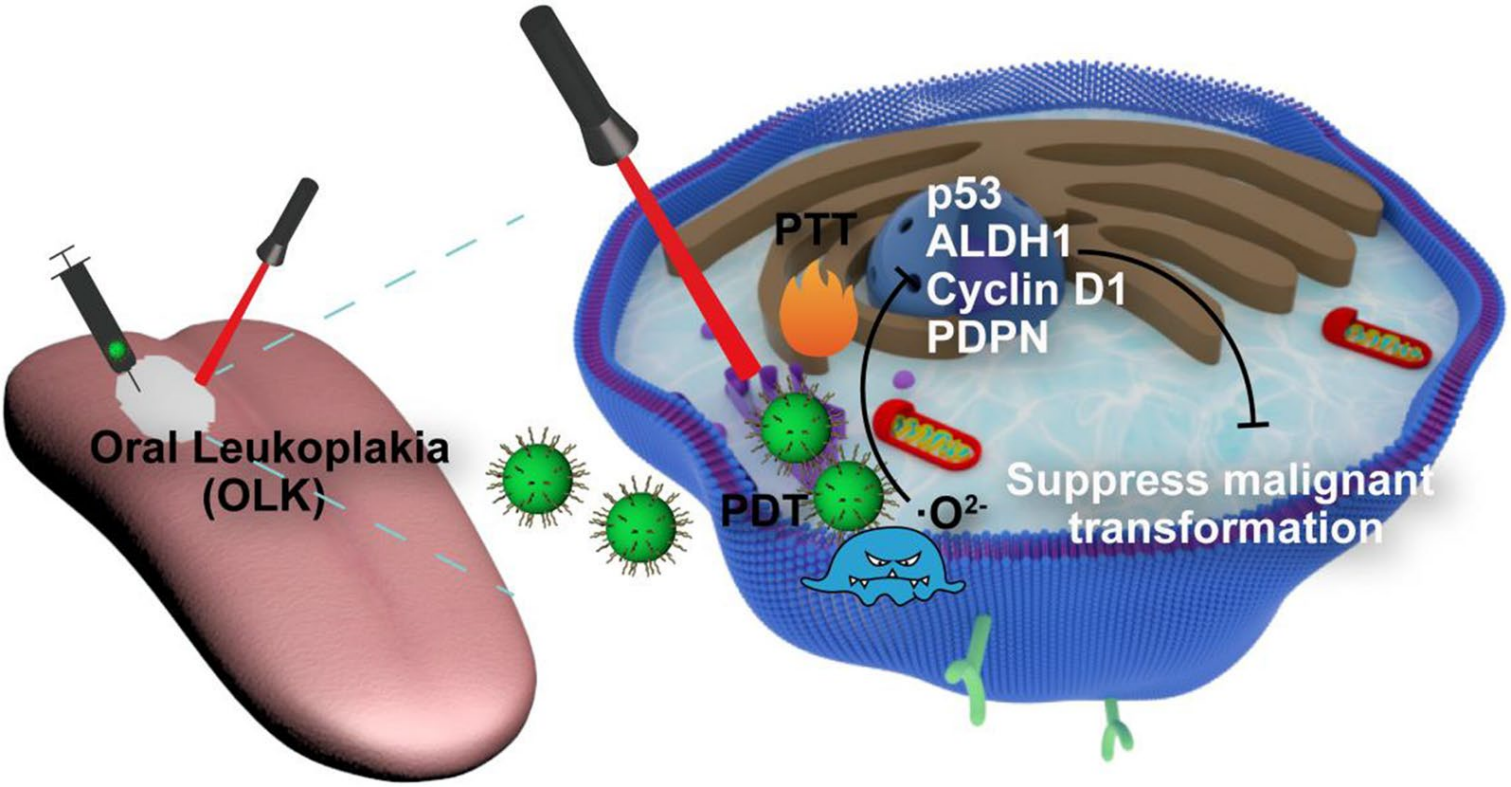
The illustration of the synergistic PDT/PTT of ITIC-Th NPs after local injection in vivo

To prevent the progression of the OPMDs to OSCC is critical to the treatment of OSCC. Local drug therapy, PDT, and surgical excision were the typical clinical therapy in the clinic, depending on different stages of OPMDs progression [14]. Local drug therapy with uncertain effects and an extended period, and the surgery is an invasive operation and full of terror to patients. The ALA-based PDT towards OLK owed the disadvantage of low response rate and complex process before and after the practice, which bring non-ideal therapeutic effect and uncomfortable feeling [15]. To conquer the practical problems, our NPs with nanoscale can work with both PDT and PTT after laser illumination via local injection, which can shorten the course of treatment and reduce painfulness if injection with anesthetics. Also, compared with ALA, the NPs can easily enter cells and further influence cell metabolism [61]. Our study showed that the tumor-relative markers were altered to a normal stage in vitro and in vivo, which ultimately induced apoptosis of OLK cells and hindered malignant transformation progression. Huge amounts of research prove that the PTT-induced cells damage can release the damage-associated molecular patterns (DAMP) [62], which can initiate the immune response in tumor therapy. Though immunotherapy in OLK is rare, its development is viable and promising, bringing new sights and possibilities in OPMDs treatment (Fig. 6).

## Conclusions

In summary, we successfully synthesized a novel organic photosensitizer ITIC-Th NPs with superior photodynamic/photothermal properties, excellent biosafety, strong anti-malignant transformation power, which was more convenient and efficient than traditional photosensitizers. The results were also exciting in premalignant animal models. As far as we know, this research was the first interdisciplinary study in the field of near-infrared fluorescence imaging and OLK. Our study provided a promising approach for photosensitizer in OLK's photodynamic and photothermal collaborative treatment, and it was of great benefit to block the canceration process of OLK, which has promising potential for future clinical treatment.

## Supplementary Information

**Additional file 1. Table S1.** Clinic-pathological features of 10 patients with oral leukoplakia. **Table S2.** Histopathological examination results of each group after PTT / PDT.**Figure S1. A** The appearance of ITIC-Th NPs in different media at room temperature after two weeks. The DLS **B** and UV-VIS spectrum **C** of NPs in pH 7.0 and pH 6.5. **Figure S2. A** Temperature change curves of ITIC-Th NPs solution ([ITIC-Th NPs] = 75 μg/mL) under 660nm laser irradiation.** B** The time constant for heat transfer was determined to be τs = 232 s. **C** The electron spin resonance (ESR) result of NPs. **Figure S3.** **A** The scheme of the experiment. **B** The near-infrared fluorescence imaging of NPs solution under different thickness covers of beef. **Figure S4.A**–**C** The cell activity assay of HaCaT, RAW264.7, and THP-1 with different concentrations of ITIC-Th NPs solutions evaluated by CCK-8. (D) Leuk-1 cells treated with hematoporphyrin for 24h, with or without laser. **Figure S5.** The ITIC-Th NPs with fluorescence in Leuk-1 and tumor cells (CAL 27) with different incubation times. Scale bar:100 μm. **Figure S6.A** The quantitative analysis of Live/Dead cells stain in Figure 4D. ***p < 0.001. **B** The quantitative analysis of Figure 4E. **p < 0.01. **Figure S7.** **A** Expression of biomarkers in representative OLK tissues was detected by immunohistochemistry (Scale bar = 100 μm). **B** Quantitative analysis of Fig A. (n = 5, mean ± SD, *p < 0.05, **p < 0.01). **C** The negative control without primary antibodies (Scale bar = 100 μm). **Figure S8.** PDT/PTT Schematic illustration of treatment in rats.

## Data Availability

All data generated or analysed during this study are included in this published article.

## Abbreviations

OLK: Oral leukoplakia
PSs: Photosensitizers
PDT: Photodynamic therapy
ITIC-Th NPs: ITIC-Th nanoparticles
PTT: Photothermal therapy
ROS: Reactive oxygen species
4NQO: 4-Nitroquinoline 1-oxide
OSCC: Oral squamous cell carcinoma
OPMDs: Oral potentially malignant disorders
WHO: World Health Organization
OLK: Oral leukoplakia
5-ALA: 5-Aminolevulinic acid
PpIX: Porphyrin IX
NIR: Near-infrared
A-D-A: Acceptor–donor-acceptor
THF: Tetrahydrofuran
ATCC: American Type Culture Collection
SOSG: Singlet Oxygen Sensor Green
DPBF: 1, 3-Diphenylisobenzofuran
CCK-8: Cell Counting Kit-8
ALDH1: Aldehyde dehydrogenase isoform 1
PDCD5: Programmed cell death 5
PDPN: Podoplanin
TEM: Transmission electron microscope
DLS: Dynamic light scattering
K-SFM: Keratinocyte serum-free medium
HaCaT: Human immortalized keratinocyte line
DMEM: Dulbecco's modified Eagle's medium
FBS: Fetal Bovine Serum
DCFH-DA: 2, 7-Dichlorodi-hydrofluorescein diacetate
SD: Sprague–Dawley
H&E: Hematoxylin and eosin
EDS: Energy-dispersive spectroscopy
ESR: Electron spin resonance
HNSCC: Head and neck squamous cell carcinoma
CDK: Cyclin-dependent kinase
CR: Complete clinical response
LN: Lymph node
