# Supplementary material for: Multifunctional photodynamic/photothermal nano-agents for the treatment of oral leukoplakia
Source: J Nanobiotechnology. 2022 Mar 4;20:106. doi: 10.1186/s12951-022-01310-2 (PMC8895861; doi:10.1186/s12951-022-01310-2)
Supplement: Supplementary file 1 — Additional file 1. Table S1. Clinic-pathological features of 10 patients with oral leukoplakia. Table S2. Histopathological examination results of each group after PTT / PDT.Figure S1. A The appearance of ITIC-Th NPs in different media at room temperature after two weeks. The DLS B and UV-VIS spectrum C of NPs in pH 7.0 and pH 6.5. Figure S2. A Temperature change curves of ITIC-Th NPs solution ([ITIC-Th NPs] = 75 μg/mL) under 660nm laser irradiation. B The time constant for heat transfer was determined to be τs = 232 s. C The electron spin resonance (ESR) result of NPs. Figure S3. A The scheme of the experiment. B The near-infrared fluorescence imaging of NPs solution under different thickness covers of beef. Figure S4.A–C The cell activity assay of HaCaT, RAW264.7, and THP-1 with different concentrations of ITIC-Th NPs solutions evaluated by CCK-8. (D) Leuk-1 cells treated with hematoporphyrin for 24h, with or without laser. Figure S5. The ITIC-Th NPs with fluorescence in Leuk-1 and tumor cells (CAL 27) with different incubation times. Scale bar:100 μm. Figure S6.A The quantitative analysis of Live/Dead cells stain in Figure 4D. ***p < 0.001. B The quantitative analysis of Figure 4E. **p < 0.01. Figure S7. A Expression of biomarkers in representative OLK tissues was detected by immunohistochemistry (Scale bar = 100 μm). B Quantitative analysis of Fig A. (n = 5, mean ± SD, *p < 0.05, **p < 0.01). C The negative control without primary antibodies (Scale bar = 100 μm). Figure S8. PDT/PTT Schematic illustration of treatment in rats. [file 12951_2022_1310_MOESM1_ESM.docx]

Multifunctional photodynamic/photothermal nano-agents for the treatment of oral leukoplakia

*Lin Lin^a,d^‡, Chuanhui Song^d,e^‡, Zheng Wei^d,f^, Huihui Zou^b,d^, Shengwei Han^b^, Zichen Cao^b,d^, Xinyu Zhang^b,d^, Guorong Zhang^d^, Jianchuan Ran^b,d^, Yu Cai*^c^, Wei Han*^b^*

*^a^Department of Oral Medicine, Nanjing Stomatological Hospital, Medical School of Nanjing University, 30 Zhongyang Road, Nanjing, 210008, China.*

*^b^Department of Oral and Maxillofacial Surgery, Nanjing Stomatological Hospital, Medical School of Nanjing University, No 30 Zhongyang Road, Nanjing, 210008, China*

*^c^Center for Rehabilitation Medicine, Rehabilitation & Sports Medicine Research Institute of Zhejiang Province, Department of Rehabilitation Medicine, Zhejiang Provincial People's Hospital（Affiliated People's Hospital, Hangzhou Medical College）, Hangzhou, Zhejiang, China 310014.*

*^d^Central Laboratory of Stomatology, Nanjing Stomatological Hospital, Medical School of Nanjing University, No 30 Zhongyang Road, Nanjing, 210008, China.*

*^e^Institute of Translational Medicine, The Affiliated Drum Tower Hospital of Nanjing University Medical School, Nanjing, 210008, China.*

*^f^Pediatric Dentistry, Nanjing Stomatology hospital, Medical school of Nanjing University, No 30 Zhongyang road, Nanjing, 210008, China.*

*E-mail: iamycai@163.com; doctorhanwei@hotmail.com.*

*‡These authors contributed equally.*

**Table S1. Clinic-pathological features of 10 patients with oral leukoplakia.**

| *Group* | *Epithelial dysplasia group (n=5)* | *%* | *Non-epithelial dysplasia group*  *(n=5)* | *%* | *P value* |
| --- | --- | --- | --- | --- | --- |
| Age (median, range) | 56±8.3 |  | 63±4.3 |  | 0.734 |
| Sex |  |  |  |  | 0.800 |
| Male | 4 | 80% | 3 | 60% |  |
| Female | 1 | 20% | 2 | 40% |  |
| Smoking |  |  |  |  | 0.738 |
| Smoker | 2 | 40% | 2 | 40% |  |
| Non-smoker | 3 | 60% | 3 | 60% |  |
| Clinical type |  |  |  |  | 0.262 |
| Non-homogenous | 4 | 80% | 2 | 40% |  |
| Homogenous | 1 | 20% | 3 | 60% |  |
| Primary site |  |  |  |  | 0.224 |
| Tongue | 2 | 40% | 2 | 40% |  |
| Palate | 2 | 40% | 0 | 0% |  |
| Buccal | 1 | 20% | 3 | 60% |  |

**Table S2. Histopathological examination results of each group after PTT / PDT.**

**Figure S1.** (A) The appearance of ITIC-Th NPs in different media at room temperature after two weeks. The DLS (B) and UV-VIS spectrum (C) of NPs in pH 7.0 and pH 6.5.

**Figure S2.** (A) Temperature change curves of ITIC-Th NPs solution ([ITIC-Th NPs] = 75 μg/mL) under 660nm laser irradiation. (B) The time constant for heat transfer was determined to be τs = 232 s. (C) The electron spin resonance (ESR) result of NPs.

**Figure S3.** (A) The scheme of the experiment. (B) The near-infrared fluorescence imaging of NPs solution under different thickness covers of beef.

**Figure S4.** (A, B, C) The cell activity assay of HaCaT, RAW264.7, and THP-1 with different concentrations of ITIC-Th NPs solutions evaluated by CCK-8. (D) Leuk-1 cells treated with hematoporphyrin for 24h, with or without laser.

**Figure S5.** The ITIC-Th NPs with fluorescence in Leuk-1 and tumor cells (CAL 27) with different incubation times. Scale bar:100 μm.

**Figure S6.** (A) The quantitative analysis of Live/Dead cells stain in Figure 4D. ***p < 0.001. (B) The quantitative analysis of Figure 4E. **p < 0.01.

**Figure S7.** (A) Expression of biomarkers in representative OLK tissues was detected by immunohistochemistry (Scale bar = 100 μm). (B) Quantitative analysis of Fig A. (n = 5, mean ± SD, *p < 0.05, **p < 0.01). (C) The negative control without primary antibodies (Scale bar = 100 μm).

**Figure S8.** PDT/PTT Schematic illustration of treatment in rats.
